# The message matters: changes to binary Computer Aided Detection recommendations affect cancer detection in low prevalence search

**DOI:** 10.1186/s41235-024-00576-4

**Published:** 2024-09-02

**Authors:** Francesca Patterson, Melina A. Kunar

**Affiliations:** Department of Psychology, The University of Warwick, Coventry, CV4 7AL UK

**Keywords:** Mammogram, Artificial intelligence, Low prevalence, Computer Aided Detection (CAD), Over-reliance, Binary CAD, Automation

## Abstract

Computer Aided Detection (CAD) has been used to help readers find cancers in mammograms. Although these automated systems have been shown to help cancer detection when accurate, the presence of CAD also leads to an over-reliance effect where miss errors and false alarms increase when the CAD system fails. Previous research investigated CAD systems which overlayed salient exogenous cues onto the image to highlight suspicious areas. These salient cues capture attention which may exacerbate the over-reliance effect. Furthermore, overlaying CAD cues directly on the mammogram occludes sections of breast tissue which may disrupt global statistics useful for cancer detection. In this study we investigated whether an over-reliance effect occurred with a binary CAD system, which instead of overlaying a CAD cue onto the mammogram, reported a message alongside the mammogram indicating the possible presence of a cancer. We manipulated the certainty of the message and whether it was presented only to indicate the presence of a cancer, or whether a message was displayed on every mammogram to state whether a cancer was present or absent. The results showed that although an over-reliance effect still occurred with binary CAD systems miss errors were reduced when the CAD message was more definitive and only presented to alert readers of a possible cancer.

## Introduction

Observers often miss rare targets in visual search at disproportionately high rates (Wolfe et al., 2005). Previous laboratory research involving participants searching for a target amongst a set of distractors suggests that as the prevalence rate of the target decreases, the proportion of miss errors (failing to notice the target) increase (e.g., Godwin et al., 2015; Hout et al., 2015; Kunar et al., 2010, 2021; Mitroff & Biggs, 2014; Rich et al., 2008; Russell & Kunar, 2012; Van Wert et al., 2009; Wolfe et al., 2005, 2007). This low prevalence (LP) effect has also shown to be robust against real-world tasks where targets are rare, such as breast screening, where radiologists inspect mammogram images for breast cancers. For example, it is estimated that 20–40% of cancers are missed in initial screening (Bird et al., 1992; see also Evans et al., 2013) and research by Evans et al. (2013) has highlighted LP as a cause of miss errors for expert mammographers in a clinical setting.^1^ Failing to detect a cancer in radiology poses serious health risks, and as such, it is vital to find ways to help improve cancer detection.

Computer Aided Detection (CAD) has been developed to aid operators in the difficult perceptual task of cancer detection by using computer algorithms to highlight suspicious features of a mammogram (Castellino, 2005). CAD typically works by overlaying a salient visual cue on the breast tissue to indicate the location of a potentially suspicious area which radiologists would then need to verify. CAD has been approved for use in radiology by the Food and Drug Administration in the USA, with a primary goal of increasing cancer detection, providing a more efficient workflow and reducing demands on radiologists (Castellino, 2005). Standard practice involves the radiologist first viewing the mammogram in the absence of CAD, then activating CAD and re-evaluating the image before issuing their final conclusion (Castellino, 2005). In laboratory studies, this reading mode has been shown to provide the optimal outcome in terms of cancer detection in comparison to conditions where readers were simultaneously presented with CAD cues on first reading of the mammogram (Kunar, 2022).

Whilst many studies have investigated the effectiveness of CAD, historically its assets and liabilities have remained controversial. For example, Lehman et al. (2015) compared the effect of CAD on digital screening mammography performance in terms of sensitivity, specificity, and cancer detection rate in a large-scale multi-screening centre study. They found that there was no improvement in screening performance when mammograms were read with CAD, compared to when CAD was not used. Furthermore, Bennett et al. (2006) conducted a literature review to compare single reading with CAD to double reading procedures (where two radiologists read the mammographic images), but differences in methodology produced indefinite conclusions. Using pooled estimates of effect sizes from two meta-analyses, Taylor and Potts (2008) found that there was no significant difference in cancer detection rates between single reading with CAD and double reading. However, differences in screening programmes used in the meta-analyses may have affected these results. While a double reading procedure remains an effective method for cancer detection (Kunar et al., 2021; Taylor & Potts, 2008), it may not be a feasible long-term approach due to the increasing number of women needing screening and the demands this place on an already limited workforce (Chen et al., 2023; Guerriero et al., 2011; James et al., 2010). Instead, recent developments in automation and Artificial Intelligence (AI) suggest that there could be improved efficacy of CAD for use in medical diagnostic imaging with systems using AI deep learning models (e.g., Fujita, 2020; Salim, et al., 2020). For example, recent research using AI as an independent supporting reader in breast cancer screening, was found to be a comparable (and in some cases superior) method to human double reading (Ng et al., 2023).

With this advancement in AI capabilities, Ng et al. (2023) also note that there is a need to evaluate new strategies for using AI technology, as supporting readers, alongside humans. Previous research has shown that the way CAD is presented to humans affects their ability to detect cancers (Kunar, 2022; Kunar & Watson, 2023). For example, it has been shown that the presence of a CAD prompt can result in an *over-reliance effect* which biases reader judgements depending on the accuracy of CAD (Kunar, 2022; Kunar & Watson, 2023; Kunar et al., 2017; Zheng et al., 2004). The over-reliance effect shows that while the use of a CAD prompt that correctly highlights a cancer decreases the amount of miss errors, there is a large increase in miss errors when the CAD system fails. For example, if the CAD cue fails to highlight a cancer or the cancer falls outside of the highlighted area, miss errors are increased in comparison to if no CAD system is used (Drew et al., 2020; Kunar et al., 2017). Furthermore, incorrect CAD cues lead to higher false alarms and subsequently recall rates where women are incorrectly recalled for further assessment (e.g., Fenton et al., 2007; Kunar, 2022; Kunar & Watson, 2023; Kunar et al., 2017). Although one could argue that miss errors, where a cancer goes undetected has potentially more serious consequences for the women involved, an increase in false alarms also has its own problems. Women who have been recalled due to a false alarm have been shown to experience psychological distress in relation to their experience (Aro, 2000) and are more likely to delay participation, or not participate at all, in future mammography screening (Kahn & Luce, 2003). Furthermore, an increase in false alarms creates unnecessary demands on healthcare systems which are already over-burdened and in crisis due to a shortage of healthcare workers (e.g., Darzi & Evans, 2016; Konstantinidis, 2023).

The over-reliance effect of using CAD is robust and is difficult to remove (although it can be mitigated in some circumstances; Kunar & Watson, 2023). One of the reasons proposed for this robust over-reliance effect is that CAD cues often use salient exogenous cues to alert the location of a cancer (Drew et al., 2020; Kunar & Watson, 2023). Exogenous cues are thought to receive higher weightings in attentional priority maps and thus capture attention automatically (Remington et al., 1992; Theeuwes, 2004; Wolfe, 2021). Even when people are told to specifically ignore these CAD cues, they still seem to elicit attentional priority leading to beneficial results when they cue the cancer, but over-reliance effects when they do not (Kunar & Watson, 2023). Having the CAD cue be a salient marker and physical presence on a mammogram may also result in other issues. For example, having a salient marker overlayed onto the mammogram may occlude parts of the breast tissue and interfere with the global regularities or ‘gist’ statistics of the mammogram. This is important as the ability to process the global image statistics of breast tissue is known to be an influential factor in detecting abnormalities in mammography (Evans et al., 2016; Raat et al., 2023).

In light of this, other CAD systems have been proposed. For example, Goldenberg and Peled (2011) discussed the advantages of CAD systems that output a simple binary recommendation of ‘positive’ or ‘negative’, indicating the presence or absence of an anomaly. Here, instead of a salient CAD cue being presented on a mammogram, a message indicating the presence or absence of a cancer would be presented elsewhere on the screen which radiologists would then have to verify (for example the message would state that a cancer is present or that a cancer is absent). This type of CAD system has recently been implemented in some AI systems within mammography where the AI system will result in a binary message to recall a woman (as a suspected cancer is present) or to not recall a woman (as either there is no cancer present or the AI has failed to detect a possible malignancy; Ng et al., 2023). CAD systems that generate binary Recall/No Recall recommendations have an advantage as they provide assistance and recommendations to human readers without the need for salient and exogenous CAD markers that are overlaid on the mammogram. However, sparse research has been conducted investigating how this type of binary message affects human decision making and in particular whether there is an over-reliance effect with this type of CAD prompt. As binary CAD systems do not use exogenous, salient cues to highlight a potential region of interest on a mammogram, then there will not be complications from over-laying a strong salient attentional cue on the breast tissue. Therefore, without these exogenous cues capturing attention the over-reliance effect previously observed with CAD markers may be reduced or eliminated. In contrast as humans are susceptible to biases, particularly in relation to recommendations given by technology (e.g., Salim Jr et al., 2023; Wysocki et al., 2023) it may be that an over-reliance effect is still observed even in the absence of these salient cues. We investigated this here.

The present study was the first investigation (at least, that the authors are aware of) into the human–computer interaction of a binary CAD system in search for a low prevalence cancer. We investigated whether users demonstrated an over-reliance on binary CAD, whereby a simple message indicated that a cancer may be present or absent. We also investigated whether there was an optimal way to present these binary CAD cues as previous research has shown that the way CAD is presented affects cancer detection. In particular, we varied the CAD messages, first, by their degree of certainty and, second, by whether the CAD message was presented only on some of the mammogram images to indicate the possible presence of a cancer or whether it was presented on every mammogram, stating that a cancer was either present or absent.

In relation to CAD certainty, it has been found that framing a CAD system to be more fallible led to a reduction in the over-reliance effect (Kunar & Watson, 2023). That is, people were more likely to perform a more exhaustive search if they were told that the CAD system was less accurate. This is particularly important in relation to search for LP targets where it is found that people often terminate their search prematurely, before they have searched the display in full (Wolfe & Van Wert, 2010). Therefore, we investigated whether manipulating the CAD message to either be definitive (i.e., a cancer *is present*—Experiments 1 and 3) or instead to be more probabilistic and less certain (i.e. a cancer *is likely*—Experiments 2 and 4) would lead to a difference in target detection.

Furthermore, we manipulated whether it was better to present CAD messages in situations where it only alerted readers to the possible presence of a cancer or whether it would be better to present messages on all mammograms to state that a cancer was either present or absent. We predicted that giving a message on some of the mammogram images versus giving a message on all mammogram images would lead to a difference in the over-reliance effect. For example, miss errors may be more pronounced under conditions where it was *explicitly* stated that a cancer was absent in comparison to when no message was shown. In this latter condition we hypothesised that, with no explicit recommendation, people would be more likely to perform an exhaustive search of the mammogram, which would result in fewer miss errors. Accordingly, in Experiments 1 and 2, we only presented the CAD message on some of the mammogram images to alert people to the possible presence of a cancer. In these experiments, if participants were shown a CAD message it was a *recall* message where we indicated the potential presence of a cancer (i.e., that a cancer was ‘present’ in Experiment 1 or ‘likely’ in Experiment 2). In a clinical setting, a ‘recall’ message would suggest indications of a possible cancer and that the woman should return to the clinic for further assessment. In contrast, a ‘no recall’ message would suggest no further assessment was needed. In Experiments 3 and 4 we presented a CAD message on *all* images of the mammograms to indicate whether a cancer was present or not. In these experiments a CAD message would either contain a *recall* or *no-recall* message (i.e., stating that a cancer was ‘present’ or ‘absent’, respectively in Experiment 3, and ‘likely’ or ‘not likely’, respectively in Experiment 4).

Throughout this study we used laboratory-based experiments where we recruited non-expert observers to search for a low prevalence simulated cancer in a mammogram. Lab-based experiments have been found to successfully examine search behaviour in applied settings (Cunningham et al., 2017; Drew et al., 2012; Drew et al., 2020; Kunar et al., 2017; Kunar et al., 2021; Kunar, 2022; Kunar & Watson, 2023; Raat et el., 2023) and are a legitimate way to test a variety of conditions that would not be otherwise feasible. Lab-based experiments using non-expert observers enable the presentation and testing of a range of CAD display options, which would not be practical in Randomised Clinical Trials (RCTs) and difficult to test with radiologists, given the demands on their time. For example, RCTs are often expensive and time-consuming. By the point of RCTs it would be important to know which are the optimal ways to present CAD and which to rule out. This information can be determined from lab-based studies, which can then be used to inform trials in a clinical setting. Furthermore, research findings that have been found to be observed in the lab have also been observed in clinical settings (Evans et al., 2013) and given that all humans share the same underlying search principles, it has been established that the search behaviour of non-expert participants is similar to those of clinicians (Drew et al., 2012; Taplin et al., 2006; Wolfe et al., 2016). Therefore, these lab-based experiments provide a valid way to investigate how binary CAD affects cancer detection.

## Method

### Transparency and openness

The data can be found on the Open Science Framework (https://osf.io/jgxz5/). All data were compiled in Microsoft® Excel® for Microsoft 365 MSO (Version 2112 16.0.14729.20254) and imported into SPSS (Version 27, Release 27 0.1.0) and JASP (Version 0.16; JASP Team, 2021) for statistical analysis. The experimental programs were written in PsychoPy (Peirce et al., 2019). The study design, hypotheses and analytic plan were not pre-registered. All manipulations, data exclusions and measures are reported.

### Participants

All participants were recruited via the University of Warwick’s Research Experience panel and received course credit as compensation for their participation in the study. A G* power analysis (alpha = 0.05, effect size = 0.5) determined that a minimum sample size of 34 participants per experiment was required to achieve a power of 0.8. Participant numbers varied slightly across experiments due to participants opting out of completing the experiment and choosing to partake in a separate activity for course credit. Thirty-nine participants took part in Experiment 1, thirty-nine participants took part in Experiment 2, thirty-eight participants took part in Experiment 3, and thirty-six participants took part in Experiment 4. None of the participants took part in more than one experiment.

### Stimuli

Stimuli included 338 images of mammograms sourced at random from the volume of 695 normal mammograms (those not containing a cancer) on the Digital Database for Screening Mammography (DDSM; Heath et al., 2001). Images were presented in the centre of a computer display and subtended approximately 10.7 degrees by 18.6 degrees at a viewing distance of 57 cm in size (please note that the actual size of each image varied as they were of real mammograms). They were categorised into ‘present’ images (an image of a cancerous mass sourced from the cancer volume of the DDSM was transposed onto the normal mammogram images using image editing software) and ‘absent’ images where no cancer was shown.

### Procedure

The experiment was created using PsychoPy, presented on a PC, and took approximately 30 min to complete. Participants were instructed to search for a cancer in the mammogram images being presented to them and were presented with example images of both mammograms containing a cancer and mammograms that did not. To familiarise participants with the task, participants were then asked to complete a training set, whereby they were presented with 10 present images and 10 absent images and were required to respond whether a cancer was present or absent via a two-alternative forced choice task and pressing the ‘m’ or ‘z’ key, respectively. Participants were required to respond correctly on 70% of the training set trials in order to continue to the experiment proper. If they failed to do so, they were given four attempts at re-completing the training block until they got (at least) 70% correct. The training block ensured that participants were able to recognise the appearance of a cancer. Following this, participants were given the experimental instructions and informed that during the experiment a cancer would be rarely present within the mammogram displays. Example images of the four trial types for that experiment were presented (manipulating both the presence of the cancer and the CAD message), followed by a practice block. Once completed, participants proceeded to the experimental block.

For each experimental block, there were 300 trials: 30 present images and 270 absent images (to give a 10% prevalence rate). For the present trials, 20 trials contained a ‘Recall’ CAD prompt explicitly indicating the presence of a cancer. The remaining 10 trials contained no CAD prompt in Experiments 1 and 2 or an explicit ‘No Recall’ CAD prompt indicating the absence of a cancer in Experiments 3 and 4. For absent trials, 180 images contained no CAD prompt in Experiments 1 and 2 or contained an explicit ‘No Recall’ CAD prompt in Experiments 3 and 4. The other 90 mammogram images contained a Recall CAD prompt. The CAD accuracy rate in these experiments was chosen to reflect CAD accuracy in a clinical setting, which is estimated to vary from 57% (Soo et al., 2005) to 85% (Obenauer et al., 2006; see also Henriksen et al., 2019, who report a CAD accuracy of between 65 and 77%). Therefore, CAD accuracy of 67% in these experiments falls within this range. Participants were not informed of the CAD accuracy rate but told that in some trials the CAD cue would give accurate information and in some trials it would not.

**Fig. 1:**
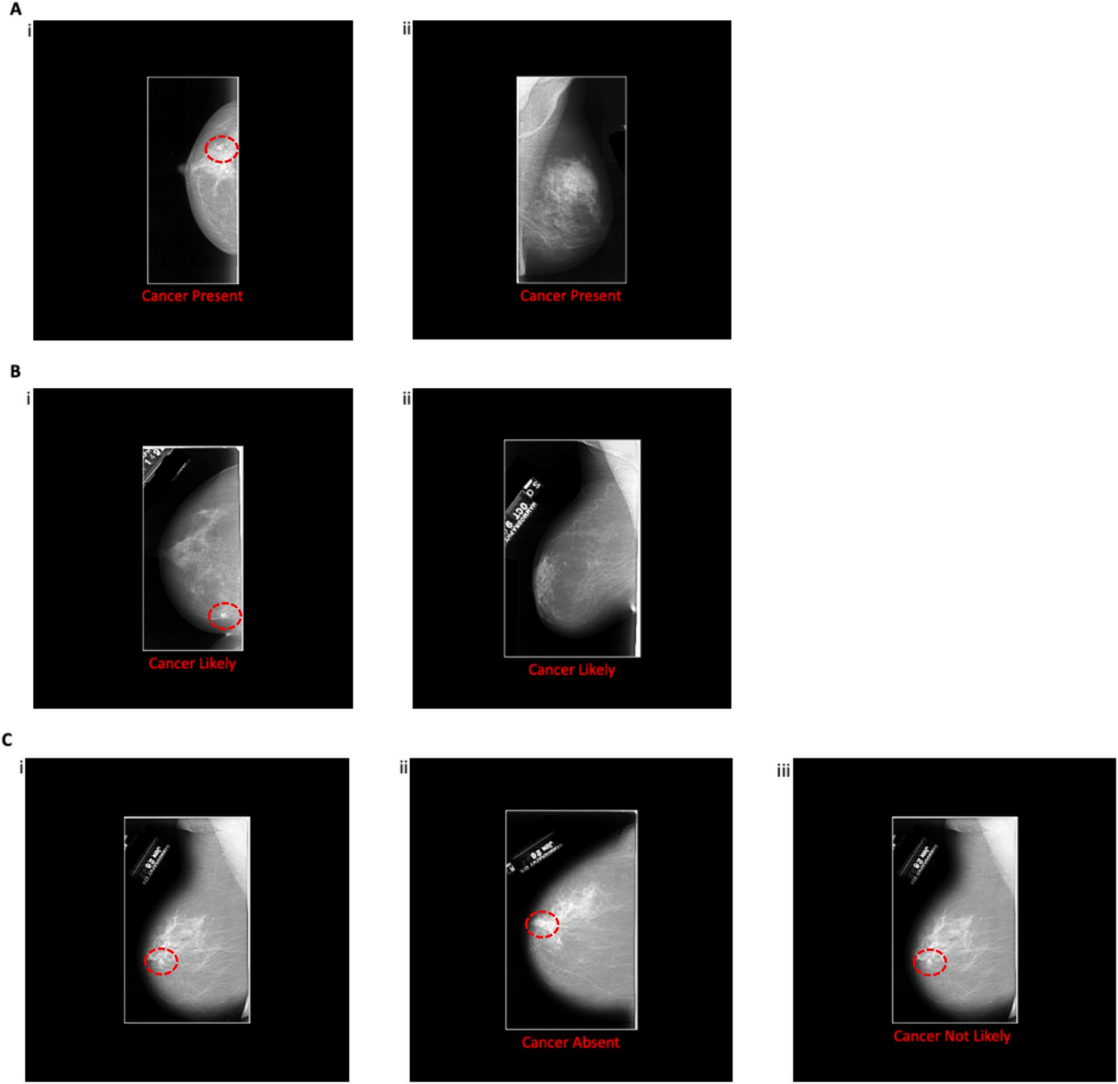
Examples of the images used in the Experiments. *Note.* Examples **A**(i) and A(ii) show a Recall CAD prompt with a ‘Cancer Present’ message (used in Experiments 1 and 3). Example **A**(i) contains a cancer, Example **A**(ii) does not. Examples **B**(i) and **B**(ii) show a Recall CAD prompt with a ‘Cancer Likely’ message (used in Experiments 2 and 4). Example **B**(i) contains a cancer, Example **B**(ii) does not. Examples C show mammograms where a cancer was present, however in the Experiments there were also images where there was no cancer. Example **C**(i) shows a No Recall trial with no CAD prompt given (used in Experiment 1 and 2). Example **C**(ii) shows a No Recall CAD prompt giving a ‘Cancer Absent’ message (used in Experiment 3). Example **C**(iii) show a No Recall CAD prompt giving a ‘Cancer Not Likely’ message (used in Experiment 4). In these examples a red dotted line highlights the position of the cancer. The red dotted line did not appear in the experiment proper

For Experiments 1 and 3 the Recall CAD prompt gave the message ‘Cancer Present’. For Experiments 2 and 4 the Recall CAD prompt showed the less definitive message of ‘Cancer Likely’. In Experiments 1 and 2 the CAD prompt (i.e., a Recall CAD message) appeared on only some of the trials. For Experiments 3 and 4 a CAD prompt appeared on all the trials. Therefore, for Experiments 1 and 2, on ‘No Recall’ trials there was no CAD prompt shown, whereas in Experiment 3, the No Recall message was ‘Cancer Absent’ and in Experiment 4, the No Recall message was ‘Cancer Not Likely’. Participants were made aware of what the CAD message would say before each experiment (e.g., ‘Cancer Present’ or ‘Cancer Likely’). Tables 1 and 2 give a summary of the different experimental conditions. Example stimuli can be found in Fig. 1.

**Table 1:** Summary of experimental conditions

Experiment	*N*	Recall—CAD message	No recall—CAD message	CAD occurrence
1	39	‘Cancer Present’	No message	Some trials
2	39	‘Cancer Likely’	No message	Some trials
3	38	‘Cancer Present’	‘Cancer Absent’	All trials
4	36	‘Cancer Likely’	‘Cancer Not Likely’	All trials

**Table 2:** Trial numbers of conditions for each experiment

	Number of recall trials	Number of no recall trials	Total number of trials
Cancer present trials	20	10	30
Cancer absent trials	90	180	270

In both the practice and experimental blocks, for each trial participants were asked to respond whether a cancer was present or absent by pressing the ‘m’ or ‘z’ key, respectively. Images were presented to each participant in a random order and remained on the screen until participants gave a response. Reaction Times (RTs) and error rates were recorded. In accordance with Fleck and Mitroff’s (2007) theory that the LP effect could be due to response-execution motor errors (where participants made motor errors by responding too fast) participants were asked to confirm their response on each trial by pressing the ‘m’ or ‘z’ key for target present and absent responses respectively. This allowed participants to self-correct any motor mistakes if they realised they had pressed the wrong key by accident. This confirmed response was used to calculate final error rates for analysis. For both the training and practice trials, feedback was provided, however none was provided on the experimental trials, mimicking conditions in a clinical setting where readers receive no immediate feedback. RTs over 10,000 ms and those less than 200 ms were considered outliers and removed from data analysis.

The over-reliance effect was measured as the difference in error rates when the CAD message indicated a cancer (i.e., in Recall trials) compared to when it did not (No Recall trials). That is, when a cancer went unprompted by CAD, were participants more likely to miss it? Furthermore, on trials where no cancer was present were participants more likely to report a false alarm with a Recall message compared to a No Recall message.

## Results

**Fig. 2:**
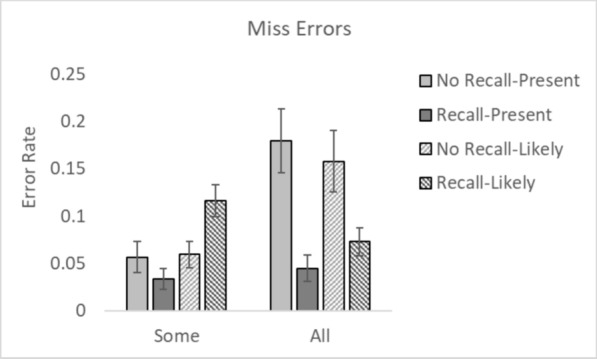
Miss error rates for all conditions. *Note.* Error bars represent the standard error

**Fig. 3:**
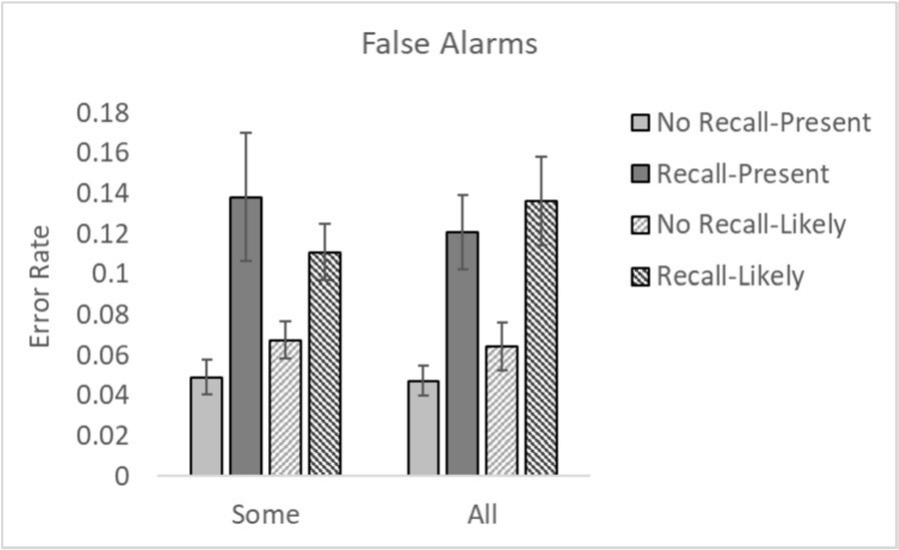
False alarm rates for all conditions. *Note.* Error bars represent the standard error

The outlier procedure removed 1.02%, 1.33%, 0.39% and 0.90% of all data in Experiments 1, 2, 3 and 4 respectively. Error rates for all conditions are presented in Figs. 2 and 3. In line with previous work, we were concerned with how CAD affected miss errors and false alarm rates independently from each other (Alberdi et al., 2004; Drew et al., 2020; Kunar, 2022; Kunar & Watson, 2023; Kunar et al., 2017). Thus, data were analysed accordingly throughout.

### Miss errors

Miss Errors were examined using a 2 × 2 × 2 repeated measures ANOVA with within participant factors of Recall (whether a CAD ‘Recall’ message was presented vs. ‘No Recall’ message) and between experiment factors of CAD Message (Present vs. Likely) and CAD Occurrence (Some vs. All trials). The results showed that there was a main effect of CAD Recall, *F*(1, 148) = 18.32, *p* < 0.001, *η*^2^_*p*_ = 0.11. Participants missed more cancers when there was a ‘No Recall’ message compared to when there was a ‘Recall’ message, showing an over-reliance effect. There was no main effect of CAD Message, *F*(1, 148) = 1.74, *p* = 0.19, *η*^2^_*p*_ = 0.01. However, the main effect of CAD Occurrence was significant, *F*(1, 148) = 7.38, *p* = 0.007, *η*^2^_*p*_ = 0.05. Participants missed more cancers in conditions where CAD was shown on all trials compared to when it was only present on some of the trials. The Recall × CAD Message interaction was significant,* F*(1, 148) = 9.03, *p* = 0.003, *η*^2^_*p*_ = 0.06, where the difference in miss errors between Recall and No Recall conditions (i.e., the 'over-reliance' effect) was greater when the CAD message said the cancer was present compared to when it was likely. The Recall × CAD Occurrence interaction was also significant, *F*(1, 148) = 34.31, *p* < 0.001, *η*^2^_*p*_ = 0.19, where the difference in miss errors between Recall and No Recall conditions (the 'over-reliance' effect) was greater when the CAD message was present on all trials in comparison to some trials. None of the other interactions were significant (all *F*s < 1.3, *p*s > 0.25).

### False alarms

A 2 × 2 × 2 repeated measures ANOVA with within participant factors of Recall (Recall vs. No Recall) and between experiment factors of CAD Message (Present vs. Likely) and CAD Occurrence (Some vs. All trials) was conducted on False Alarms. There was a main effect of CAD Recall, *F*(1, 148) = 58.75, *p* < 0.001, *η*^2^_*p*_ = 0.28. Participants made more false alarms when the CAD prompt indicated the presence of a cancer, compared to when it did not (or when no CAD prompt was given). There was no main effect of CAD Message, *F*(1, 148) = 0.16, *p* = 0.69, *η*^2^_*p*_ = 0.001. Neither was there a main effect of CAD Occurrence, *F*(1, 148) = 0.002, *p* = 0.96, *η*^2^_*p*_ = 0.000. None of the interactions were significant (all *F*s < 1.8, *p*s > 0.19).

### Signal detection theory

**Fig. 4:**
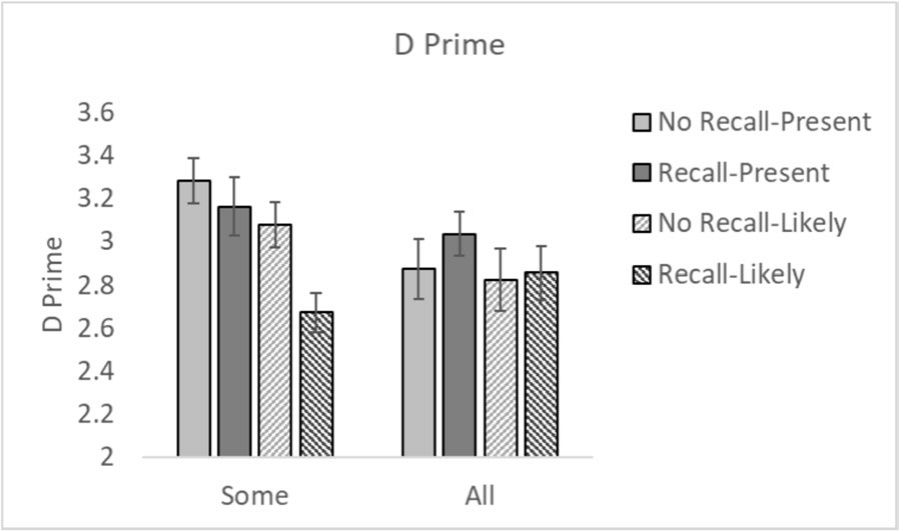
D’ values for all conditions. *Note.* Error bars represent the standard error

**Fig. 5:**
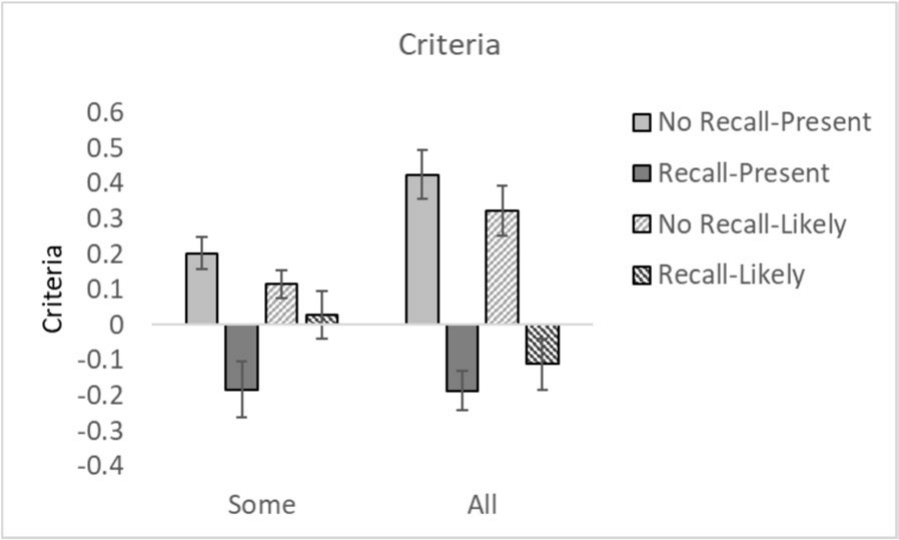
C values for all conditions. *Note.* Error bars represent the standard error

Signal Detection Theory (SDT, Green & Swets, 1966; Macmillan & Creelman, 2005) was used to calculate *d’* (sensitivity) and *c* (criterion) in each experiment.^2^ Figures 4 and 5 shows the *d’* and *c* values, respectively.

### Sensitivity (*d’*)

A 2 × 2 × 2 repeated measures ANOVA with within participant factors of Recall (Recall vs. No Recall) and between experiment factors of CAD Message (Present vs. Likely) and CAD Occurrence (Some vs. All trials) was conducted on *d’*. There was no main effect of Recall, *F*(1, 148) = 2.76, *p* = 0.10, *η*^2^_*p*_ = 0.018. Neither was there a main effect of CAD Occurrence, *F*(1, 148) = 1.99, *p* = 0.16, *η*^2^_*p*_ = 0.013. However, there was a main effect of CAD Message, *F*(1, 148) = 4.63, *p* = 0.03, *η*^2^_*p*_ = 0.03. D Prime was greater when the CAD message stated that a cancer was ‘present’ in comparison to when it was ‘likely’. There was a significant Recall × CAD Message interaction, *F*(1, 148) = 4.31, *p* = 0.04, *η*^2^_*p*_ = 0.03, in which the difference in *d’* between a Recall and No Recall CAD was greater when the CAD Message indicated a cancer was likely rather than a cancer was present. There was also a significant Recall x CAD Occurrence interaction, *F*(1, 148) = 13.28, *p* < 0.001, *η*^2^_*p*_ = 0.082, in which the difference in *d’* between the Recall and No Recall conditions was greater when the CAD message was shown on some of the trials versus when it was shown on all of the trials. None of the other interactions were significant (all *F*s < 1.2, *p*s > 0.28).

### Criteria, (*c*)

A 2 × 2 × 2 repeated measures ANOVA with within participant factors of Recall (Recall vs. No Recall) and between experiment factors of CAD Message (Present vs. Likely) and CAD Occurrence (Some vs. All trials) was conducted on *c*. There was a main effect of Recall, *F*(1, 148) = 176.74, *p* < 0.001, *η*^2^_*p*_ = 0.54, in which participants were less willing to respond that a target was present in the No Recall condition in comparison to the Recall condition. There was no main effect of CAD Occurrence, *F*(1, 148) = 1.60, *p* = 0.21, *η*^2^_*p*_ = 0.011. Neither was there a main effect of CAD Message, *F*(1, 148) = 0.18, *p* = 0.67, *η*^2^_*p*_ = 0.001. There was a significant Recall × CAD Message interaction, *F*(1, 148) = 17.58, *p* < 0.001, *η*^2^_*p*_ = 0.11, in which there was a bigger difference in response criteria between the Recall and No Recall condition when the CAD message said the cancer was present compared to when it said the target was likely. There was also a significant Recall × CAD Occurrence interaction, *F*(1, 148) = 25,14, *p* < 0.001, *η*^2^_*p*_ = 0.15, in which there was a bigger difference in response criteria between the Recall and No Recall condition when the CAD message was presented on all trials compared to some of the trials. None of the other interactions were significant (all *F*s < 1.2, *p*s > 0.29).

### Comparison of CAD systems: which system is best?

**Fig. 6:**
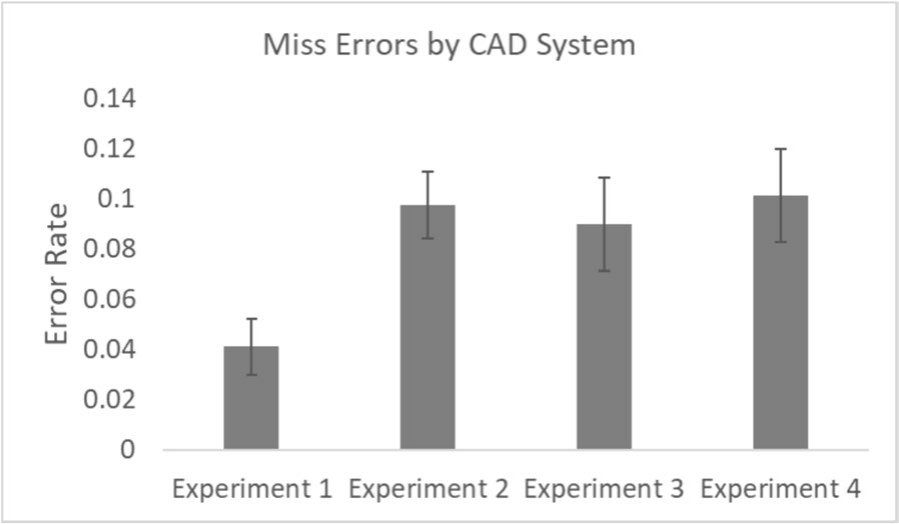
Overall mean miss errors across CAD systems in experiments 1–4. *Note.* Error bars represent the standard error

**Fig. 7:**
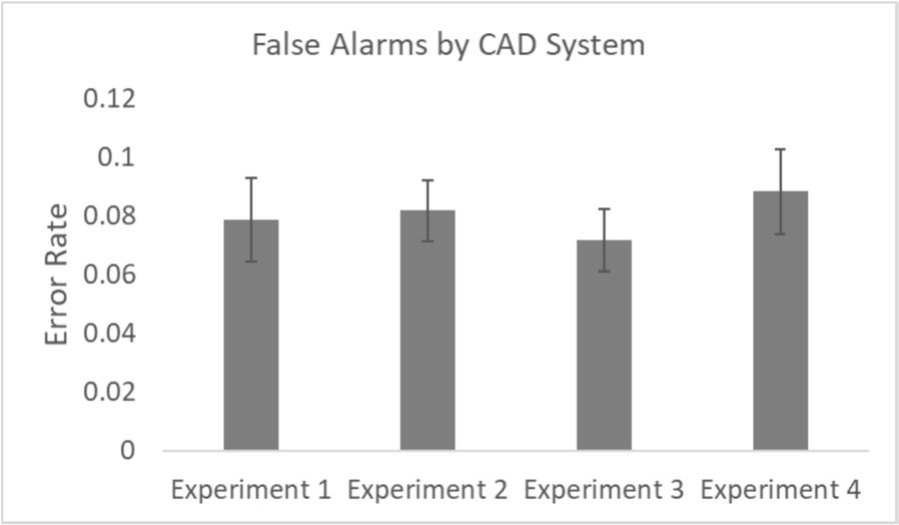
Overall mean false alarms across CAD systems in experiments 1–4. *Note.* Error bars represent the standard error

To compare CAD Systems across the experiments we calculated the mean overall miss errors, false alarms, the Recall Rate and the Positive Predictive Value (PPV) for Experiments 1–4 (see Figs. 6, 7 and Table 3). Recall Rate (i.e., the percentage of mammograms that were reported to have abnormal findings) and PPV (i.e., the percentage of women recalled for further tests who have cancer) are important clinical metrics within breast cancer screening (e.g., Norsuddin et al., 2015; Rauscher et al., 2021; Taylor-Phillips et al., 2024) and were calculated as follows, in which TP stands for True Positive, FP stands for False Positive (false alarms), TN stands for True Negative and FN stands for False Negative:

$${\text{Recall}}\;{\text{Rate}} = \frac{{\sum \left( {{\text{TP}} + {\text{FP}}} \right)}}{{\sum \left( {{\text{TP}} + {\text{FP}} + {\text{TN}} + {\text{FN}}} \right)}} \times 100$$

$${\text{PPV}} = \frac{{\sum {\text{TP}}}}{{\sum \left( {{\text{TP}} + {\text{FP}}} \right)}} \times 100$$

**Table 3:** Mean recall rate and PPV across experiments

	Recall rate (%)	PPV (%)
Experiment 1	16.7 (1.30)	67.6 (3.44)
Experiment 2	16.5 (0.98)	61.0 (2.80)
Experiment 3	15.5 (0.99)	64.6 (3.03)
Experiment 4	16.9 (1.34)	61.6 (3.52)

Standard errors are shown in the parentheses

One way between experiment ANOVAs were used to analyse which of the CAD systems (if any) showed better performance in each of these measures. The results showed there to be a difference in the miss errors across CAD systems, *F*(3, 148) = 3.22, *p* = 0.02, *η*^2^_*p*_ = 0.061, with the CAD system in Experiment 1 producing fewer overall miss errors compared to the other systems. However, there was no difference across CAD systems in false alarms, *F*(3, 148) = 0.29, *p* = 0.83, *η*^2^_*p*_ = 0.006, Recall Rates, *F*(3, 148) = 0.27, *p* = 0.85, *η*^2^_*p*_ = 0.006, or PPV,^3^*F*(3, 148) = 0.68, *p* = 0.56, *η*^2^_*p*_ = 0.014.

## General discussion

Previous research has shown that when a CAD system used exogenous cues to highlight a cancer, an over-reliance effect emerged where participants became overly dependent on the CAD cues. The current study investigated whether an over-reliance effect also occurred with CAD systems that presented binary CAD recommendations alongside the mammogram. Experiments 1 and 2 presented a CAD message on some of the trials to indicate the presence of a cancer. In Experiment 1 the message stated that a cancer was present, while in Experiment 2, the message stated that a cancer was likely. Experiments 3 and 4 presented a CAD message on every trial to indicate that a cancer was either present or absent (Experiment 3) or that a cancer was either likely or not likely (Experiment 4).

The data make several important points. First, even when using CAD as a binary system, an over-reliance effect emerged. For False Alarms, participants were more likely to (incorrectly) respond that a cancer was present when shown a CAD Recall message. For miss errors, participants were more likely to miss a cancer when there was no CAD message (Experiments 1 and 2) or when they were presented with a No Recall message (Experiments 3 and 4). This over-reliance effect was greater when a CAD message was presented on *all trials* compared to when it was only presented on *some* of the trials. Furthermore, the over-reliance effect was more pronounced when the CAD message said the cancer was ‘present’ compared to when it said a cancer was ‘likely’. In a clinical setting, any increase in miss errors and false alarms have their own associated problems. Miss errors are obviously worrying as it means that an undiagnosed cancer will go untreated, having potentially serious health consequences for the women involved. False alarms frequently mean that recalled women undergo further tests which can be both invasive and costly (in terms of time and money) to both the women involved and the healthcare system. Furthermore, women who have been falsely recalled have been known to report feelings of psychological distress (Aro, 2000) and may delay participation or not participate at all in future screening programs (Kahn & Luce, 2003).

Please note that in Experiment 2, miss errors when a Recall message was present were higher than trials when there was no CAD message. This pattern was opposite of what would be predicted from the over-reliance effect. The reason for this was unclear. One could argue that the message ‘Cancer Likely’ led people to ‘second guess’ and dismiss the CAD cue more often compared to when the CAD cue was definitive (Cancer Present). However, this seems unlikely given that the same pattern was not observed in Experiment 4. It may be that miss errors in Experiment 2 were artificially inflated in this experiment, but as the mammogram stimuli were identical across experiments it is again unclear what would be driving this. Future research will be needed to investigate this further.

A comparison between binary CAD systems showed that while there was no difference in overall false alarms, Recall Rate or PPV across experiments, there were fewer miss errors for the CAD system tested in Experiment 1. Miss errors are an important metric that can be measured in the laboratory but cannot be easily determined in a clinical setting as by definition, a radiologist will only become aware that they have missed a cancer if the woman becomes symptomatic between routine breast screening checks. An explanation for the reduction in miss errors in Experiment 1 may be gleaned from the SDT data, which showed that participants’ sensitivity to detect a target was greater when the CAD message read ‘present’ rather than ‘likely’. Furthermore, on No Recall trials participants were less willing to commit to a response that a cancer was present when the CAD was shown on all trials, versus some of them. Based on these experiments we would recommend the binary CAD system in Experiment 1 be tested for use in a clinical setting (e.g., definitive messages presented only on mammograms where a cancer is suspected). Future research would, of course, be needed to ascertain whether the same benefits would occur with medical readers. Despite this, there is compelling evidence to show that principles found in the lab can be applied to healthcare professionals. For example, the proportion of miss error rates in the lab are similar to radiologists reading mammograms in a clinical setting (Evans et al., 2013). Furthermore, over-reliance effects with salient CAD cues, also appear to occur with radiologists (Zheng et al., 2004). Given that similar search strategies have been found in both non-medical and medically-trained readers (Wolfe et al., 2016) we would predict that a similar benefit found in the binary CAD system of Experiment 1 would also be likely to occur to breast cancer screening in the real world.

Please also note that the over-reliance effect observed in these binary CAD systems may be affected by radiologist experience. It has been suggested that radiologists with more experience tend to interact with CAD less than those with less experience (Hupse et al., 2013). Goldenberg and Peled (2011) suggested that for binary CAD systems, outcomes that are positive in identifying a disease should be verified by more experienced readers, while those with a negative outcome could be considered by less experienced staff (particularly if CAD systems were used as a way to triage patients). However, we would suggest that for mammography, dividing cases based on staff experience would not result in best practice. Triaging acute medical conditions based on CAD outputs would be important in emergency situations where diagnosis is time critical as urgent cases could be treated by an experienced physician in a timely manner (Goldenberg & Peled, 2011). However, in cases where the disease is chronic, such as with cancer screening, the benefit of separating CAD outputs via reader experience would be negligible and possibly damaging. That is, if less experienced clinicians were more dependent on CAD, they would be more likely to miss a cancer if it was not flagged by the CAD prompt. Thus, consideration of how to allocate CAD outputs across readers with different experience needs to be considered by future research.

It is also worth noting that mammogram reading procedures differ globally. Double reading mammogram procedures are considered standard practice in the UK, (Chen et al., 2023) and across most other European countries (Balta et al., 2020), whereas single reading is the more common practice in the USA. The use of CAD in mammogram screening across different countries will therefore be affected by current practices and regulations in different parts of the world. Nevertheless, as double reading is considered labour intensive and there is continued concern about the number of radiologists currently available (Chen et al., 2023) there has been increased interest as to whether AI and automated aids can be feasibly used as a ‘second reader’ to help workflow within mammographic screening (Geras et al., 2019; Rodriguez-Ruiz et al., 2019). Data from experiments such as these can help inform clinicians of the optimal way to present AI prompts to readers.

There are, of course, limits to the conclusions that we can make based on this study given that the experimental procedure is very different to clinical procedures in mammography. First, in our experiments participants were only shown one mammogram image at a time with no control over how the image was presented. This is very different from normal clinical practice in which radiologists have a custom hanging protocol for how images are displayed. Furthermore, radiologists are able to view images from prior mammograms for comparison if they chose to whereas, participants were not offered that option in our experiments. This may have affected participant’s over-reliance on the CAD prompt, as they had less control of what they were presented. Second, in Experiments 3 and 4 a CAD message was shown on all trials. For present purposes, we have compared errors in the Recall vs. No Recall conditions to measure the over-reliance effect. However, in future work it would be good to compare these data to a baseline condition in which No CAD message was presented. Third, in a clinical setting the prevalence rate of a cancer is much lower than the 10% prevalence rates exhibited in our experiments. Wolfe et al. (2005) have found that miss errors increase as the prevalence of the target decreases. Furthermore, search strategies change under low prevalence conditions. Wolfe and Van Wert (2010) proposed a Multiple-Decision Model stating that at low prevalence, the time spent searching a display before terminating search is decreased and there is also a shift in response bias so that people require more evidence before committing to a target present response. Further research would be needed to determine if the effects found in the current study also apply to a clinical setting when searching for a cancer with a lower prevalence rate.

CAD use in mammography has the potential to help save lives. The rise of AI in breast screening is promising in terms of helping readers with the mammography task and helping with the workload in healthcare systems. Of course, along with the visual output of CAD recommendations it is also important to consider where to add AI into the healthcare workflow. Having AI be fully automated and used as a standalone reader to make a clinical recommendation would be beneficial in relieving the workload for healthcare professionals (Raya-Povedano et al., 2021). However, it has been found participants feel more comfortable with AI acting as an additional reader in the workflow, acting alongside a human rather than it being used to make an independent clinical diagnosis outside of human input (Ng et al., 2023; Ongena et al., 2021). Ng et al. (2023) have suggested that adding AI into the workflow as a *supporting* reader may be the best approach to capture the benefits of CAD, while assuaging the concerns of patients. Here, the AI acts as a second reader. However, in situations where there is a disagreement between the human reader and the AI then the case is deferred to another human reader for assessment. Given the range of possibilities of presenting CAD and how the different strategies affect clinical outcomes it is clear that ever-more research is needed to investigate how humans optimally interact with CAD systems.

## Conclusion

Previous research has found that over-trust in computer-aided systems can lead users to make diagnostic errors (Jorritsma et al., 2015). The present data add to the narrative that the way we present CAD to humans affects how we interact with it. Although Goldenberg and Peled (2011) hoped that binary CAD would be an advancement of traditional CAD systems, our work has shown that binary CAD systems are still susceptible to over-reliance behaviours. Showing that a similar effect also occurs in a clinical field will be important to establish going forward with the increased exploration of how AI and other automated decision systems will be rolled out across healthcare services.

## Data Availability

The datasets generated and/or analysed during the current study are available in the Open Science Framework repository, https://osf.io/jgxz5/.

## Abbreviations

LP: Low prevalence
CAD: Computer Aided Detection
ANOVA: Analysis of variance
RTs: Reaction Times
USA: United States of America
AI: Artificial Intelligence
DDSM: Digital Database for Screening Mammography
RCT: Randomised Clinical Trial

## Footnotes

1 This relates to routine screening mammography, for women who have no signs or symptoms of breast cancer. The prevalence of breast cancer is higher in symptomatic screening of mammograms.

2 False alarm or miss error rates of 0 and 1 were adjusted using the formulas 1/2*n* and 1 − (1/2*n*), where *n* = the number of trials (Macmillan & Kaplan, 1985, see also Russell & Kunar, 2012; Wolfe et al., 2007; Kunar et al., 2021; Kunar, 2020; Kunar & Watson, 2023, who used this procedure).

3 Please note that, although the PPV rates found in these experiments were similar to PPV rates found in Europe, they are higher than those typically found in the USA (which range from 15 to 30% approximately; Kopans, 1992). These differences are likely to be due to these experiments being run in a laboratory compared to a clinical setting. Further research would be needed to investigate how Recall Rates and PPV are affected by binary CAD presentation within a real-world setting.
